# Editorial: Crosstalk between peripheral and local immune response in the pathophysiology of stroke and neurodegeneration diseases

**DOI:** 10.3389/fncel.2023.1270834

**Published:** 2023-09-05

**Authors:** Yuanjian Fang, Anwen Shao, Lei Huang, Jiping Tang, Xiangsheng Zhang, Dirk M. Hermann

**Affiliations:** ^1^Department of Neurosurgery, The Second Affiliated Hospital, School of Medicine, Zhejiang University, Hangzhou, Zhejiang, China; ^2^Department of Neurosurgery, Loma Linda University, Loma Linda, CA, United States; ^3^Department of Physiology and Pharmacology, Loma Linda University, Loma Linda, CA, United States; ^4^Department of Anesthesiology, Loma Linda University, Loma Linda, CA, United States; ^5^Department of Neurosurgery, Beijing Friendship Hospital, Capital Medical University, Beijing, China; ^6^Department of Neurology, University Hospital Essen, University of Duisburg-Essen, Essen, Germany

**Keywords:** immune response, neurodegeneration, stroke, local, peripheral inflammation

The role of immune response in neurological disease pathophysiology continues to be a hot topic in recent decades. Accumulating evidence reveals both local and peripheral immune systems contribute to in stroke, traumatic brain injury, and neurodegenerative diseases (Waisman et al., 2015). Immune cell activation is also involved through facilitating inflammatory responses in Alzheimer's disease, Parkinson's disease, and amyotrophic lateral sclerosis. Despite the central nervous system (CNS) being described as an immune-privileged site, both local and peripheral immune cells participate in the immune response in the progression of these neurodegenerative diseases (Waisman et al., 2015). In the CNS parenchyma, two glia cells, astrocytes and microglia are actively involved in the local innate immune response. In contrast, the peripheral lymphocytes like T cells, B cells and master cells, monocytes, can cross the blood-brain barrier (BBB) contributing to immune activation (Greenhalgh et al., 2020; Harms et al., 2021). However, both innate and adaptive immune responses in CNS disorders are double-edged swords, which not only promote repair and recovery but also accelerate brain injury.

Neuroimmunologists seek to understand the interactions between the local innate immune response and peripheral adaptive immune response both under homeostatic conditions and in diseases (Kipnis, 2016). In pathological conditions, peripheral immune cells, cytokines, and complements can cross the BBB to cause direct neurotoxicity that contributes to microglia and astrocyte activation. Additionally, local immune cell activation can also release cytokines and chemokines to attract neutrophils and initiate local inflammatory responses. Immune responses have broad implications for with various other pathological changes, such as BBB disruption, oxidative stress, and programmed cell death. Multiple signaling pathways are activated by the stimulation of local and peripheral immune responses which further modifies the function of non-immune cells such as endothelial cells, neurons, pericytes, and oligodendrocytes (Kipnis, 2016). A better knowledge of the roles of both peripheral and local immune responses in stroke and neurodegenerative diseases may provide new therapeutic opportunities to explore neuronal rescue and repair strategies.

The topic of “*Crosstalk between peripheral and local immune response in the pathophysiology of stroke and neurodegenerative diseases*,” has attracted many original research and review articles, including papers on molecular and cellular mechanisms, as well as clinical translational applications. A total of 11 reviews and 18 original articles were collected in this topic, studying stroke (Chen H. et al.), spinal cord injury (Chen, Shen et al.; Tang et al.), glioma (Lin et al.), Alzheimer's disease (AD) (Chen, Dai et al.), Parkinson's disease (PD) (Zhang Z. et al.), traumatic brain injury (TBI) (Wang et al.) and pediatric septic shock (Fan et al.).

For stroke, Cheng et al. and Fu et al. summarized the interaction between peripheral and local immunity, as well as the role of autophagy in immune responses after stroke onset and the potential immunotherapeutic strategies. In addition, Zhong et al. and Tuz et al. reviewed and summarized the effect of sex and gastrointestinal function in stroke progression, the role of estrogen as an immunomodulator in immune reactions, and the potential clinical value of estrogen replacement therapy, which may help better understand the immunomodulatory function and provide a basis for its novel therapeutic use in stroke. Meanwhile, Zhao et al. used bibliometric analysis of tumor necrosis factor (TNF) in post-stroke neuroinflammation from 2003 to 2021, revealing the important role of TNF in post-stroke neuroinflammation which may represent a potential target for neuroprotective therapeutics after stroke.

Several original studies revealed new therapeutic targets for subarachnoid hemorrhage (SAH), including salvianolic acid B, gasdermin D (GSDMD), pleckstrin homology-like domain family A member 1 (PHLDA1), and sestrin2. Salvianolic acid B ameliorates neuroinflammation and neuronal injury via blocking NOD-like receptor thermal protein domain associated protein 3 (NLRP3) inflammasome and promoting silent mating type information regulation 2 homolog - 1 (SIRT1) after SAH in rats (Zhao et al., 2021). A newly discovered GSDMD inhibitor, LDC7559 was found to suppress neuronal pyroptosis and microglial activation after experimental SAH (Cai et al.). Similarly, PHLDA1 blockade was found to modulate microglial responses and NLRP3 inflammasome signaling following experimental SAH (Lai et al.). Both endogenous and exogenous nuclear factor erythroid 2-related factor 2 (Nrf2) promoted microglia polarization from the M1 to M2 phenotype after SAH in rats (Yang et al.). Interestingly, Han et al. found inhibition of Axl reduced macrophage polarization toward the M1 phenotype via the STAT1/HIF-1α signaling pathway and prevented aneurysm SAH to happen in mice.

Meanwhile, Su et al. found that γδ T cells recruitment and local proliferation in brain parenchyma alleviated neuroinflammation after micro-intracerebral hemorrhage (ICH). Zhang L. et al. used spatial transcriptome analysis to discover hematoma-stimulated circuits for secondary brain injury after intraventricular hemorrhage, which may help design more effective clinical management regimens and develop novel bioinformatics strategies for the study of other CNS diseases as well. Luo et al. investigated the neutrophil-to-lymphocyte ratio (NLR), platelet-to-lymphocyte ratio (PLR), and D-dimer-to-fibrinogen ratio (DFR) as predictors of pneumonia and poor outcomes in patients with ICH.

Sun et al. retrospectively reviewed 741 consecutive patients with acute ischemic stroke who underwent intravenous thrombolysis with rtPA and found that platelet-to-lymphocyte ratio at 24h after thrombolysis is a prognostic marker in acute ischemic stroke patients. Mu et al. explored two datasets (GSE37587 and GSE16561) from the Gene Expression Omnibus database and found 6 critical genes, STAT3, FPR1, AQP9, SELL, MMP9, and IRAK3, related to oxidative stress and neutrophil response in early ischemic stroke, which may provide new insights into understanding the pathophysiological mechanisms of ischemic stroke. Shen et al. revealed human umbilical cord mesenchymal stem cells (hUCMSCs) could inhibit the infiltration of inflammatory cells in the brain tissue and promote the repair of brain tissue structure and function. Early intervention by injecting high-dose of hUCMSCs could significantly improve the recovery of neurological/motor function and reduce the size of cerebral infarction in rats. Additionally, Zhang Q. et al. found electroacupuncture (EA) and induced pluripotent stem cell (iPSC)-derived small extracellular vesicles (iPSC-EVs) treatments regulated intestinal immunity through MGBA regulation of intestinal microbes, reducing brain and colon damage following cerebral ischemia and positively impacting the outcomes of ischemic stroke.

For neurodegenerative diseases, Zhang Z. et al. discussed the structural function of toll-like receptors and signal transduction in Parkinson's disease by reviewing clinical studies, animal models, and *in vitro* studies, which highlighted the therapeutic value of toll-like receptors in immune therapies of PD. Huang's review concluded that positron emission tomography (PET) imaging of neuroinflammation is a promising approach to decipher the enigma of the pathophysiological process of AD and mild cognitive impairment. Interestingly, Chen, Dai et al. first revealed activated biological markers leukocyte cell adhesion molecule and vascular cell adhesion molecule-1 as indicators of AD. Houser et al. also found progranulin and Glycoprotein Nonmetastatic Melanoma Protein B to jointly regulate the peripheral and the central immune system in a sex-dependent manner, which may also play a role in neurodegenerative diseases.

These studies enriched our understanding of immune responses in the pathophysiology of neurological diseases. We extend our deepest gratitude to all the contributing authors and reviewers who participated in this Research Topic. And we are looking forward to more reviews and original articles covering to the new Research Topic “*Crosstalk between peripheral and local immune response in the pathophysiology of stroke and neurodegenerative diseases, volume II*”.

## Author contributions

YF: Writing—original draft. AS: Writing—review and editing. LH: Conceptualization, Writing—review and editing. JT: Supervision, Visualization, Writing—review and editing. XZ: Writing—original draft. DH: Supervision, Project administration, Writing—review and editing.

## References

[B1] Greenhalgh A. D. David S. Bennett F. C. (2020). Immune cell regulation of glia during CNS injury and disease. Nat. Rev. Neurosci. 21, 139–152. 10.1038/s41583-020-0263-9 32042145

[B2] Harms A. S. Ferreira S. A. Romero-Ramos M. (2021). Periphery and brain, innate and adaptive immunity in Parkinson's disease. Acta. Neuropathol. 141, 527–545. 10.1007/s00401-021-02268-5 33555429 PMC7952334

[B3] Kipnis J. (2016). Multifaceted interactions between adaptive immunity and the central nervous system. Science 353, 766–771. 10.1126/science.aag2638 27540163 PMC5590839

[B4] Waisman A. Liblau R. S. Becher B. (2015). Innate and adaptive immune responses in the CNS. Lancet Neurol. 14, 945–955. 10.1016/S1474-4422(15)00141-6 26293566

[B5] Zhao Y. Zhang Y. Zhang J. Yang G. (2021). Salvianolic acid B protects against MPP^+^-induced neuronal injury via repressing oxidative stress and restoring mitochondrial function. Neuroreport 32, 815–823. 10.1097/WNR.0000000000001660 33994527

